# Supplementary material for: Human Milk Archaea Associated with Neonatal Gut Colonization and Its Co-Occurrence with Bacteria
Source: Microorganisms. 2025 Jan 4;13(1):85. doi: 10.3390/microorganisms13010085 (PMC11767358; doi:10.3390/microorganisms13010085)
Supplement: Supplementary file 1 [file microorganisms-13-00085-s001.zip › Salas-Lopez_Microorganisms-Supplementary Materials-v05.pdf]

microorganisms
MDPI

Article

Human milk archaea associated with neonatal gut colonization and its co-occurrence with bacteria.

Maricarmen Salas-López [1], Juan Manuel Vélez-Ixta [1], Diana Laura Rojas-Guerrero [1,2], Alberto Piña-Escobedo [1], José Manuel Hernández-Hernández [1], Martín Noé Rangel-Calvillo [3], Claudia Pérez-Cruz [4], Karina Corona-Cervantes [1,5], Carmen Josefina Juárez-Castelán [1,*] and Jaime García-Mena [1,*]

[1] Departamento de Genética y Biología Molecular, Cinvestav, Av. Instituto Politécnico Nacional 2508, Mexico City 07360, Mexico; maricarmen.salas@cinvestav.mx (M.S.-L.); juan.velez@cinvestav.mx (J.M.V.-I.); diana.rojas@cinvestav.mx or dianarogue@hotmail.com (D.L.R.-G.); apinae@cinvestav.mx (A.P.-E.); jose.hernandezh@cinvestav.mx (J.M.H.-H.); karina.corona@cinvestav.mx or karina.corona333@gmail.com (K.C.-C.)
[2] Institute of Environmental Sciences, Faculty of Biology, Jagiellonian University, Gronostajowa 7, 31-007 Kraków, Poland
[3] Hospital General "Dr. José María Rodríguez", Ecatepec de Morelos 55200, Mexico; drrangelcalvillo@gmail.com
[4] Departamento de Farmacología, Cinvestav, Av. Instituto Politécnico Nacional 2508, Mexico City 07360, Mexico; cperezc@cinvestav.mx
[5] Institute for Obesity Research, Monterrey Institute of Technology and Higher Education, Monterrey 64849, Mexico
* Correspondence: carmen.juarez@cinvestav.mx (C.J.J.-C.); jgmena@cinvestav.mx (J.G.-M.)

Supplementary Materials

1. Supplementary Tables

Table S1. Sequencing summary

Parameter	Colostrum (n = 40)	Neonatal stool (n = 40)
Number of reads	2,269,715	2,261,733
Mean of reads per sample	56,743	56,543
Standard deviation	30,844	30,610
Standard error mean	4,877	4,840
Median of reads	49,434	47,553
Lowest sample reads	16,980	4,573
Highest sample reads	161,512	123,102
Median length for sequences	259 bases	259 bases

Table S2. Decontam results. Analyses were performed using negative control shown in Figure S3, prevalence method was used with a p threshold of < 0.1 No contaminant was found according to decontam.

(please find the table attached as an Excel file Salas-López et al-Microorganisms-01-Table S2_decontam_results).

Table S3. Summary of numerical values of Phyla Relative Abundances (%)

Phylum	Sample type	mean ± SD	median ± IQR
Halobacteriota	Colostrum	88.21 ± 16.88	93.07 ± 16.37
	Neonatal stool	89.53 ± 21.58	97.34 ± 11.59
Methanobacteriota_A_1229	Colostrum	11.79 ± 16.88	6.93 ± 16.37
	Neonatal stool	10.47 ± 21.58	2.66 ± 11.59

SD, Standard Deviation; IQR, Interquartile range.

Table S4. Summary of numerical values of relative abundances of genera

Genus	Sample type	mean ± SD	median ± IQR
*Methanoculleus*_A_2118	Colostrum	52.61 ± 26.14	52.83 ± 34.71
	Neonatal stool	52.12 ± 24.49	51.89 ± 27.45
*Methanosarcina*_2619	Colostrum	35.53 ± 20.16	40.39 ± 28.19
	Neonatal stool	37.30 ± 22.48	39.80 ± 27.7
*Methanobrevibacter*_A	Colostrum	5.74 ± 16.83	0.00 ± 3.17
	Neonatal stool	5.45 ± 21.57	0.00 ± 0.00
*Methanothermobacter*_A_884	Colostrum	6.05 ± 7.14	3.70 ± 10.48
	Neonatal stool	5.02 ± 7.31	0.73 ± 8.27
Methanofollis	Colostrum	0.07 ± 0.34	0.00 ± 0.00
	Neonatal stool	0.11 ± 0.37	0.00 ± 0.00

SD, Standard Deviation, IQR, Interquartile range.

Table S5. Statistical test for Alpha Diversity Indexes

Index	Effect	Test	Statistic	*p*-value	*q*-value*
Observed	0.42	KW	3.66	0.06	0.13
Shannon	0.49	KW	4.10	0.04	0.13
Simpson	0.41	KW	4.68	0.03	0.12
InvSimpson	0.55	AOV	5.89	0.02	0.09
Fisher	0.47	KW	4.10	0.04	0.13

KW, Kruskall-Wallis; AOV, Analysis of Variance; *, with Holm correction.

2. Supplementary Figures

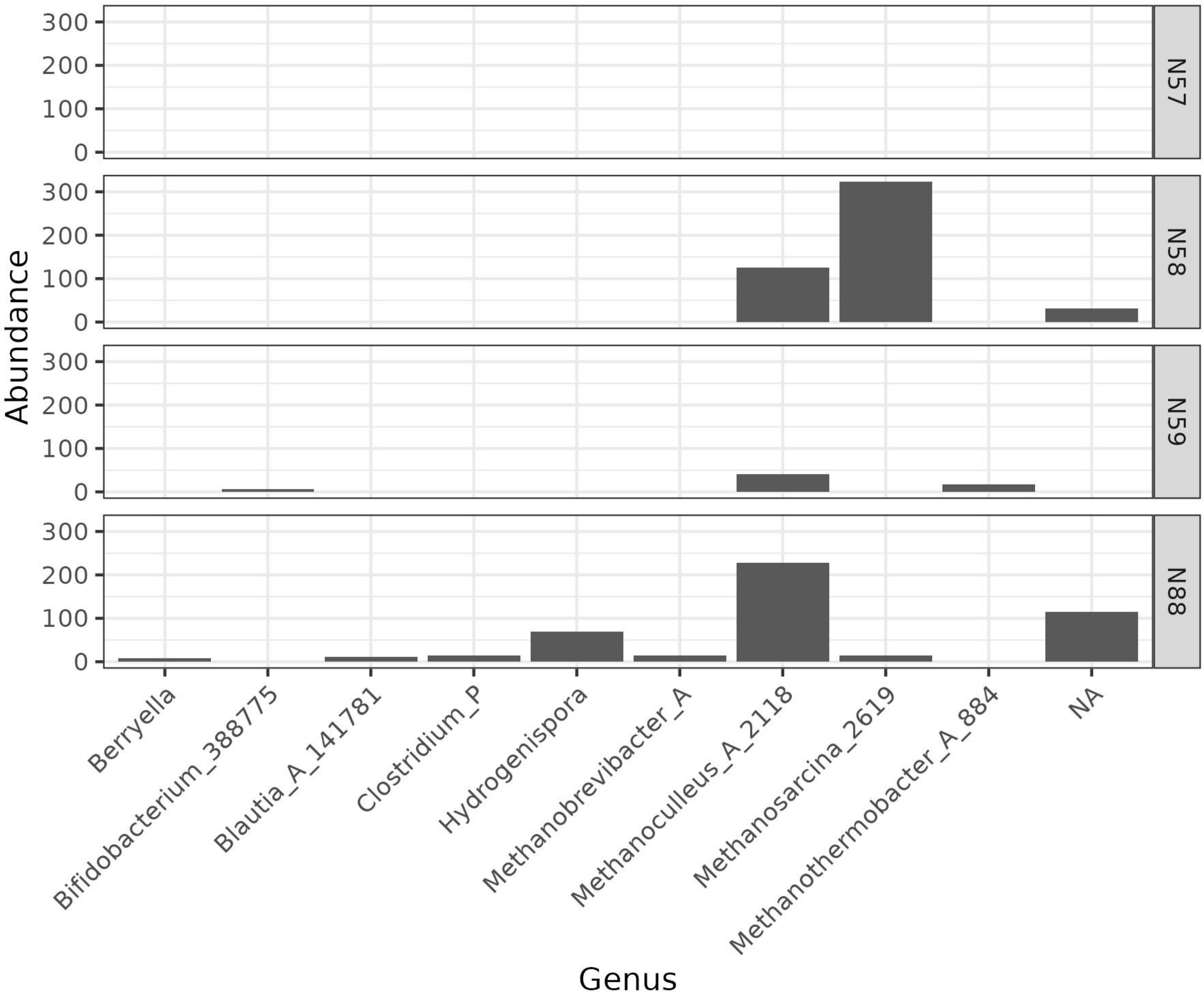

Figure S1. Sequencing results of archaeal and bacterial abundance reads in negative controls used for Decontam. It can be noted that one of the controls showed no reads (N57). The most abundant ASVs in negative controls were identified as *Methanoculleus* and *Methanosarcina*. *Methanoculleus* had more than 100 reads only in N58 and N88 negative controls, while *Methanosarcina* had the highest number of reads (> 300), but this only happened in N58. However, when performing the Decontam none of our ASVs corresponded to contaminants. X-axis bars indicate the taxa, while Y-axis indicates how many times (counts) the taxa were found.

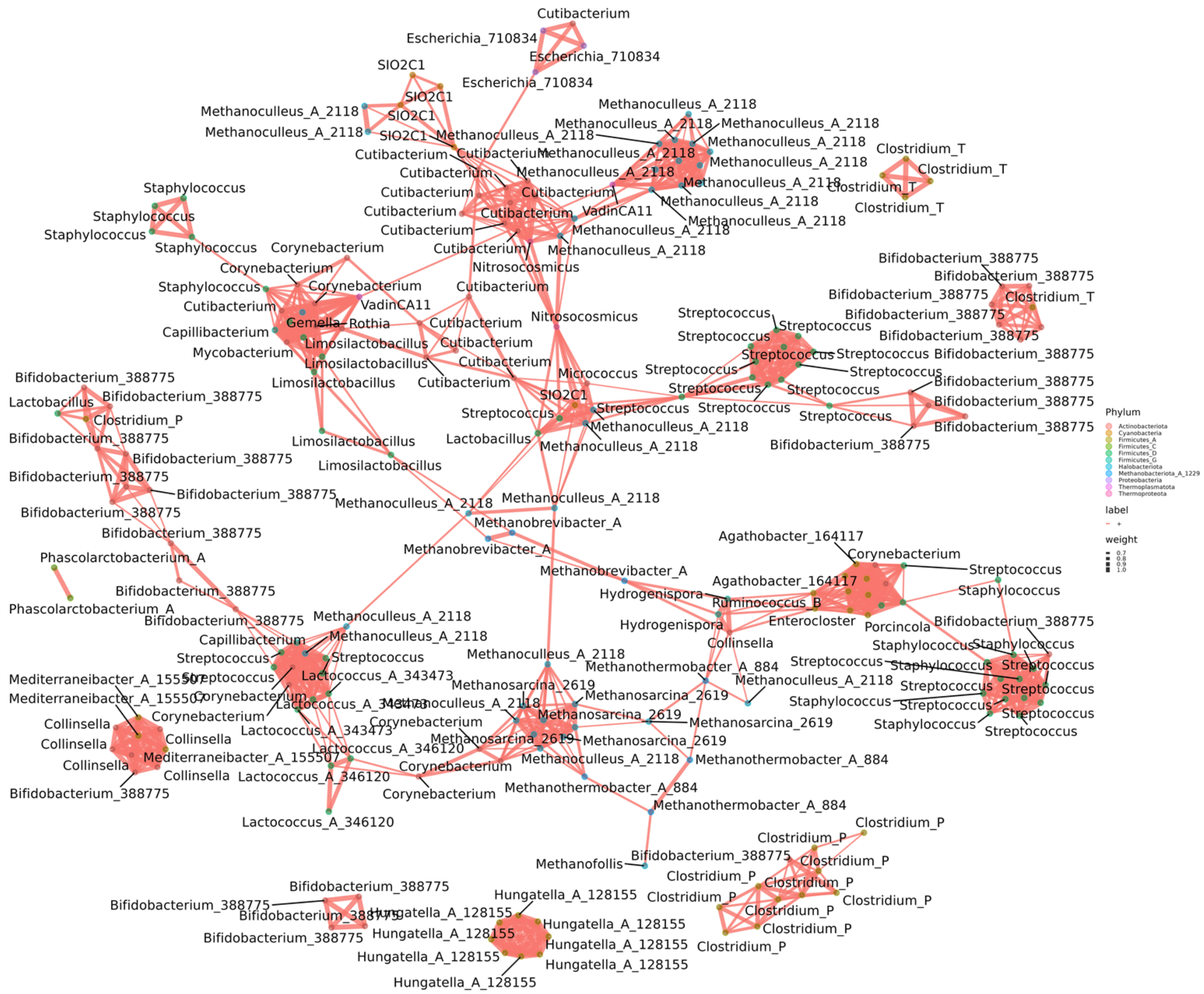

Figure S2. Microbial co-occurrence network in the neonatal stool. Nodes are represented as colored dots, edges as links connecting nodes. The color in the nodes indicates Phylum. A connection between nodes stands for a strong (Spearman's $\rho > 0.6$) and significant ($p > 0.01$) positive correlation. Labels indicate Genus.

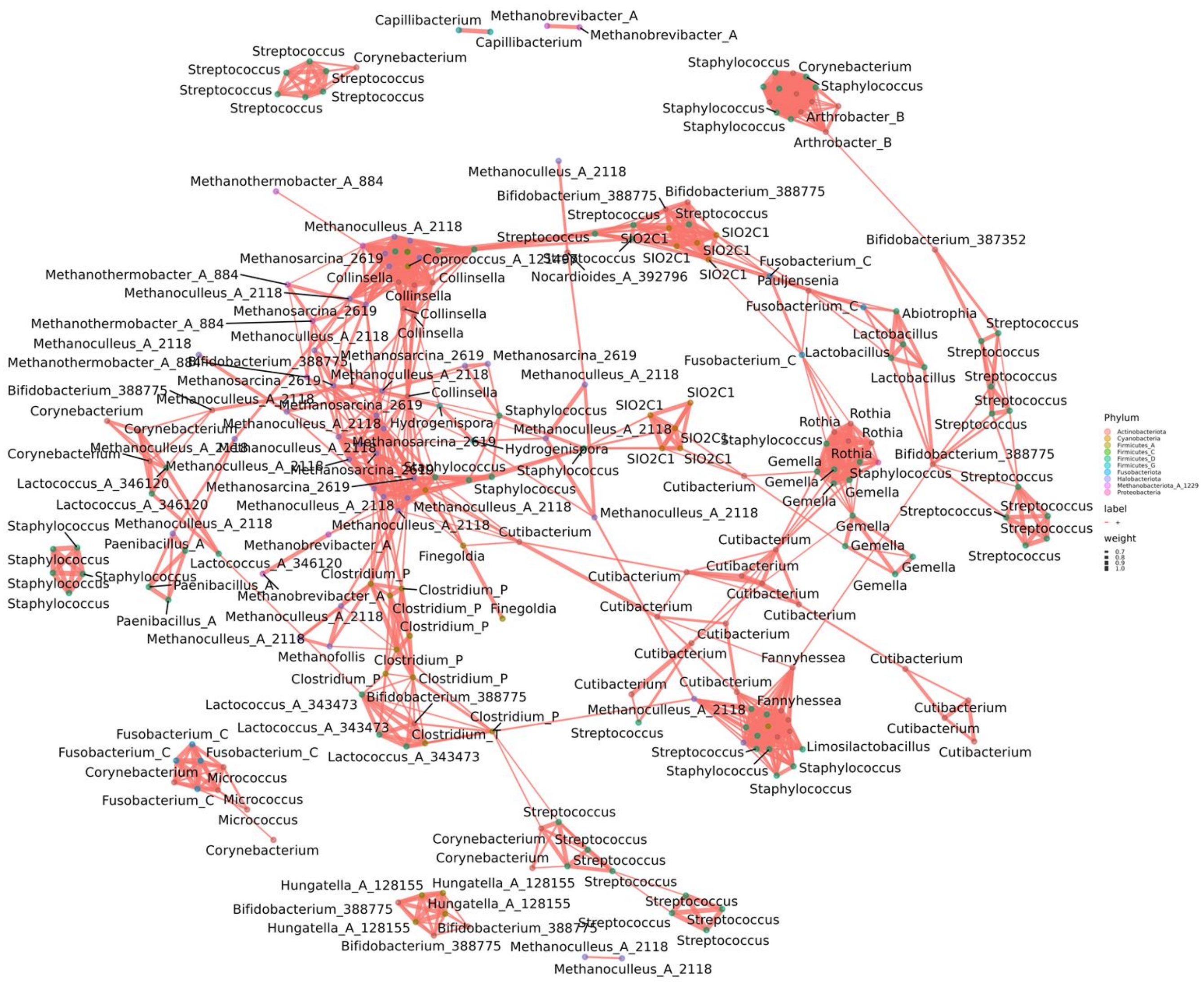

Figure S3. Microbial co-occurrence network comparison in human colostrum. Nodes are represented as colored dots, edges as links connecting nodes. The color in the nodes indicates Phylum. A connection between nodes stands for a strong (Spearman's $\rho > 0.6$) and significant ($p > 0.01$) positive correlation. Labels indicate Genus.

--end-of-text--
